# Draft Genome Sequence of the Antibiotic-Producing Epiphytic Isolate *Pantoea ananatis* BRT175

**DOI:** 10.1128/genomeA.00902-13

**Published:** 2013-11-07

**Authors:** Derek D. N. Smith, Morgan W. B. Kirzinger, John Stavrinides

**Affiliations:** Department of Biology, University of Regina, Regina, Saskatchewan, Canada

## Abstract

*Pantoea* is a member of the *Enterobacteriaceae*, whose members have been shown to produce novel antibiotics. Here, we report the 4.8-Mb genome sequence of *Pantoea ananatis* strain BRT175, an epiphytic isolate from strawberries that produces an antibiotic that is effective against the fire blight pathogen, *Erwinia amylovora*.

## GENOME ANNOUNCEMENT

*Pantoea ananatis* is a highly diverse species of the *Enterobacteriaceae*. It is a notable plant pathogen, causing disease in maize (1), eucalyptus (2, 3), onions (4–6), and rice (3), but it is also an opportunistic human pathogen, causing subcutaneous and systemic infections in adults and children (7, 8). Many isolates of *P. ananatis* have been shown to have beneficial properties, such as the plant growth-promoting *P. ananatis* B1-9, which was shown to enhance yields of red pepper crops 3-fold (9), and *P. ananatis* CPA-3, which was shown to be an effective agent for postharvest control of fungi, like *Penicillium expansum* (10).

Several *Pantoea* species have been shown to produce a variety of antimicrobials, such as pantocins (11–14), herbicolins (15, 16), microcins (17–19), and phenazines (20), several of which target amino acid biosynthesis genes in the fire blight pathogen, *Erwinia amylovora* (21–24). Several antibiotic-producing isolates, including *Pantoea agglomerans* E325 and *Pantoea vagans* C9-1, have been developed into biocontrol agents and are registered for use against *E. amylovora* (25). Here, we report the genome sequence of an epiphytic isolate, *P. ananatis* BRT175, which produces a highly potent novel antibiotic that is effective against *E. amylovora*.

Genome sequencing was performed using Illumina HiSeq 2000 with 100 bp paired-end sequencing. The 16,222,954 reads with an average Phred quality score of 32 were assembled *de novo* using ABySS version 1.3.5 (26), using an optimized *k*-mer value of 81. This resulted in 89 contigs with an N_50_ of 363,457 bp and an estimated genome size of 4,851,883 bp at 334× coverage. Contigs of ≥200 bp (43 total) were submitted to the NCBI Prokaryotic Genome Automatic Annotation Pipeline version 2.0, resulting in 4,696 predicted genes, consisting of 4,563 coding sequences, 38 pseudogenes, 21 rRNAs, and 74 tRNAs. One contig appears to represent a plasmid that is 160,004 bp in size.

The genome sequence of *P. ananatis* BRT175 not only provides the means for identifying useful natural products but also yields important genomic information for uncovering the genetic basis for human opportunism in *P. ananatis.*

### Nucleotide sequence accession numbers.

This whole-genome shotgun project has been deposited at GenBank under the accession no. ASJH00000000. The version described in this paper is the first version, ASJH01000000.
